# Evolution of wing scales in Diptera documented by fossils

**DOI:** 10.1186/s40851-024-00244-x

**Published:** 2024-12-30

**Authors:** Ewa Krzemińska, Wiesław Krzemiński, Iwona Kania-Kłosok, Jadwiga Stanek-Tarkowska, Kornelia Skibińska, Daubian Santos

**Affiliations:** 1 Institute of Systematics and Evolution of Animals, Polish Academy of Sciences, Sławkowska 17, Kraków, 31-016 Poland; 2 Department of Biology, University of Rzeszów, Zelwerowicza 4, Rzeszów, 35–601 Poland; 3 Institute of Agricultural Sciences, Land Management and Environmental Protection, University of Rzeszów, Zelwerowicza 8B, Rzeszów, 35-601 Poland; 4 Centro de Ciências Naturais e Humanas, Universidade Federal do ABC, Av. dos Estados, 5001. Bairro Bangu, Santo André, SP 09210-580 Brazil

**Keywords:** *Maietta*, New species, Limoniidae, Baltic amber, Paleoclimate

## Abstract

Among the insects with wings clad in scales, the butterflies are the best known and those showing greatest variety of scale types. In the Diptera, some families or particular genera of two large groups are known to bear scales on wings, i.e., mosquitoes (Culicomorpha) and moth flies (Psychodomorpha). From among another large dipteran group, the crane-flies (Tipulomorpha), scales are present on wings only in one small genus, *Maietta* Alexander, now endemic to the southwestern coast of South America. Here, we describe an Eocene ancestor of *Maietta*, embedded in Baltic amber, *Maietta hoffeinsetta*, n. sp. This species and its recent congeners document evolution of scale cover from sparse and scarce, restricted only to anterior portion of wing, to complete and dense. A similar parallel evolutionary route was previously described in the Culicidae. The fossil representative of *Maietta* provides also a further example of biogeographical relationships of Baltic fauna with recent congeners distributed today far from Europe. The present finding prompts a discussion on a possible role of scales in adaptation to post Eocene cooling down of climate.

**Supplementary Information:**

The online version contains supplementary material available at 10.1186/s40851-024-00244-x.

## Background

Insect wings usually bear specialized hairs, the micro- and macrotrichia. In butterflies (Lepidoptera) and some flies (Diptera), a third type of superficial structure, scales, also appears on the wings. Scales are conspicuous and remarkable feature in butterflies and usually cover an entire wing, arranged in overlapping rows. The amazing abundance of scale shapes and colours, and their role in sexual selection in butterflies is widely known [1, 2]. In this insect order, scales exhibit extremelycomplex shapes [3] and sophisticated properties, such as variegated angles of reflection [4, 5], or differential UV reflection in conspecific males and females [6]. Other roles of lepidopteran scales include acoustic camouflage against bats in moths [7], thermoregulation [8, 9], predator avoidance and mimicry [10], and pheromone distribution by specialized (androconial) scales [11].

Much less is known about dipteran scales. These occur only in some families or separate genera; the mosquitoes and allies (infraorder Culicomorpha), and more rarely in moth flies (Psychodomorpha) and gall midges (Cecidomyiidae; Bibionomorpha). Dipteran scales are similar to those of the Lepidoptera in their basic form, which is a narrow petiole anchored in the wing membrane and a broader, flat portion thatelaborates into longitudinal ridges interconnected by thinner crossribs. The shape of the distal portion varies from narrow and lanceolate to spatula-like; multiple scale shapes are often encountered on the same wing [12]. The functional role of scales in the Diptera remains rather poorly known. Hairs and scales help cleaning the wing and make it less wettable, as water drops slide on the hairs and fall off together with dirt particles [13] (however, notably no functional distinction is made here between the hairs and the scales). In some species of mosquitoes, the scales are bi-colored; dark scales among lighter ones may form a spot pattern on wings, which is likely related to sexual recognition, as such patterns are diagnostic to species [14].

Scales evolved from the macrotrichia, and the development of both structures is mediated by the same gene in Lepidoptera and Diptera [15, 16]; in the Diptera, transient forms between macrotrichia and scales can be observed (Culicidae [17], other Diptera [18]). Diptera and Lepidoptera belong to a single large group, the Mecopterida, and represent crown taxa of its two large sister groups: the Antliophora and the Amphiesmenoptera, respectively [19]. In more recently diverged taxa, scales are absent from wings in remaining members of the Mecopterida, i.e., the scorpion flies (Mecoptera) and fleas (Siphonaptera) within the Antliophora, and caddis flies (Trichoptera) within the Amphiesmenoptera.

Fossil evidence shows, however, that scales were present in extinct basal members of the Amphiesmenoptera, i.e., in order Tarachoptera [20] and family Lepidochlamidae, a stem group of the Trichoptera [19]. The Triassic age of these insects is inferred from their phylogeny [21], as both the Tarachoptera and Lepidochlamidae were described from Cretaceous Burmese amber (99 Mya). The oldest imprints of loose scales come from the beginning of the Triassic (Hettangian [22] and are attributed to butterflies. Preserved lepidopteran wings with scales are known from as early as the early Jurassic [23, 24]. All butterflies embedded in Cretaceous and younger resins have wings covered with scales of various shapes, e.g [25]. , . Usually, these are small micro-moths (Micropterigidae), only a few milimeters in length (e.g., inclusions in Burmese amber [26] and Baltic amber [27]).

From among the Antliophora, only the Diptera and their separated families and genera, possess wing scales wing; interestingly, scales have never been identified in the Mecoptera, which is a lineage of Permian origin, ancestral to the Diptera. Thus, dipteran scales seem to represent a remote echo of the common genetic ancestry of the Mecopterida reaching back probably at least to the early Triassic, given that the oldest representatives of aforementioned insect groups are not known prior to this epoch (Diptera [28]; Lepidoptera [22]; Trichoptera [24]; Tarachoptera [20]. Unlike the Amphiesmenoptera, scales in Diptera are not known before the Cretaceous; the oldest scales were recorded from Burmese amber and belong to a mosquito [17, 29]. The stepwise evolution of the scale wing cover in the Culicomorpha is well documented [17, 29–33]; however, no fossil representative of any other dipteran group with scales has been reported, including among those in which more recent taxa do bear scales on the wings (e.g., the Psychodidae).

Here, we present the first fossil of a crane fly (Tipulomorpha), in which the wings are partially covered with scales. This fossil represents an extinct congener of the only genus of this infraorder with scaled wings, *Maietta* Alexander, 1929. This genus of six species is now endemic to Chile [34]. An Eocene ancestor of this peculiar genus was found embedded in Baltic amber, which indicates the much wider distribution of this species in the past and provides a further example of the biogeographical relationships among Baltic fauna with more recent congeners now distributed far from Europe. This species and its recent congeners document the evolution of scale cover from scarce and limited in coverage only to the costal portion of wing and the fringe margin, to complete and dense. This new finding prompts discussion on possible roles for scales in adaptation to post-Eocene climatic cooling .

## Materials and methods

### Materials

All studied specimens have been deposited in public institutions. The presentstudy was based on an inclusion in Baltic amber (age: Eocene, Lutetian–Priabonian [35] from the private collection of Christel and Hans Werner Hoffeins. The holotype described herein is deposited in the Senckenberg Deutsches Entomologisches Institut (SDEI), Müncheberg, Germany.

Comparative recent specimens: *Maietta trimedia* Alexander, 1967, Chile, Aysen, 25 km S of Cochuane, 1–2.02.1990, coll. L. E. Peña, housed in the Institute of Systematics and Evolution of Animals, Polish Academy of Sciences, Kraków, Poland (ISEA PAS), and the specimens of this species listed in [34].

### Methods

Specimens were examined using a Nikon (SMZ25) stereomicroscope equipped with a Nikon digital camera (DS-Ri2) at the ISEA PAS, photographs and drawings were made using a Nikon SMZ 1500 stereomicroscope equipped with a Nikon DS–Fi1 camera at the University of Rzeszów, Poland. Usually, 10–30 stacked photographs were combined by use of Helicon Focus 5.3 × 64 (Helicon Soft Ltd (c) 2013). Measurements were taken using NIS–Elements D 3.0 software. Drawings were completed by tracing the photographs. Terminology of morphological structures: wing venation [28], male genitalia [36].

Bodies of *Maietta trimedia* Alexander, and a mosquito, *Ochlerotatus* sp., were analyzed under a Hitachi SU 8010 scanning electron microscope (SEM) at the Podkarpacie Innovative Research Center of the Environment (PIRCE) at the University of Rzeszów. For SEM observations, specimens were attached to aluminum stubs and sputtered with 20 nm of gold using a Turbo-Pumped Sputter Coater Quorum Q 150OT ES.

## Results

### Systematic paleontology

Order Diptera Linnaeus, 1758.

Infraorder Tipulomorpha Rohdendorf, 1961.

Family: Limoniidae Speiser, 1909.

Subfamily: Chioneinae Rondani, 1841.

Genus: *Maietta* Alexander, 1929.

*Maietta* Alexander, 1929: 184 [37]. 

*Maietta hoffeinsetta* Krzemiński, Krzemińska & Santos, n. sp., urn:lsid:zoobank.org:pub:FD4DF571-A20B-44C3-A10C-69AD3120FF7C.

#### Etymology

The name of new species refers to Christel and Hans Werner Hoffeins, who acquired this remarkable inclusion and generously made it available for study by our group.

#### Differential diagnosis

In *M*. *hoffeinsetta*, n. sp., costal and subcostal sectors of wing covered with oval, petiolate scales, which are also present on legs; the remainder of the wings are covered with numerous long macrotrichia (in recent species of *Maietta*, the entire wing is covered with scales). Antennae twice as long as palpi, filiform; flagellomeres (16) thin apart from three basal ones, which are oval (in recent *Maietta*, antennae as short as head, flagellomeres rounded); palpi with third segment subtriangular, expanded, and fourth twice longer than all preceding combined (in recent *Maietta*, third segment not enlarged; fourth segment somewhat exceeding combined length of three preceding ones). Thorax: large katepisternum, anepimeron and meron, all three of comparable size and shape in entire genus. Tibial spurs absent. Wing venation same as in recent *Maietta*: Sc half as long as wing; R2 (r-r) arched, relatively long; discal cell absent; M1 and M2 fused into M1 + 2 over entire length; m-cu long. Male genitalia in fossil and recent species: gonocoxite with acute distal extension on ventral side; gonostyle probably two-branched, outer branch fleshy, little shorter than gonostyle, inner branch in shape of narrow sickle. Female unknown (in recent species of *Maietta* cerci of ovipositor short, round).

#### Holotype

No. HT♂, CCHH #1651-2 / SDEI = Dip-00436 - Baltic amber, Ch. and H.W. Hoffeins collection, deposited in the Senckenberg Deutsches Entomologisches Institut (SDEI), Müncheberg, Germany. Age: Eocene, Lutetian–Priabonian [35].

#### Description

**Fig. 1:**
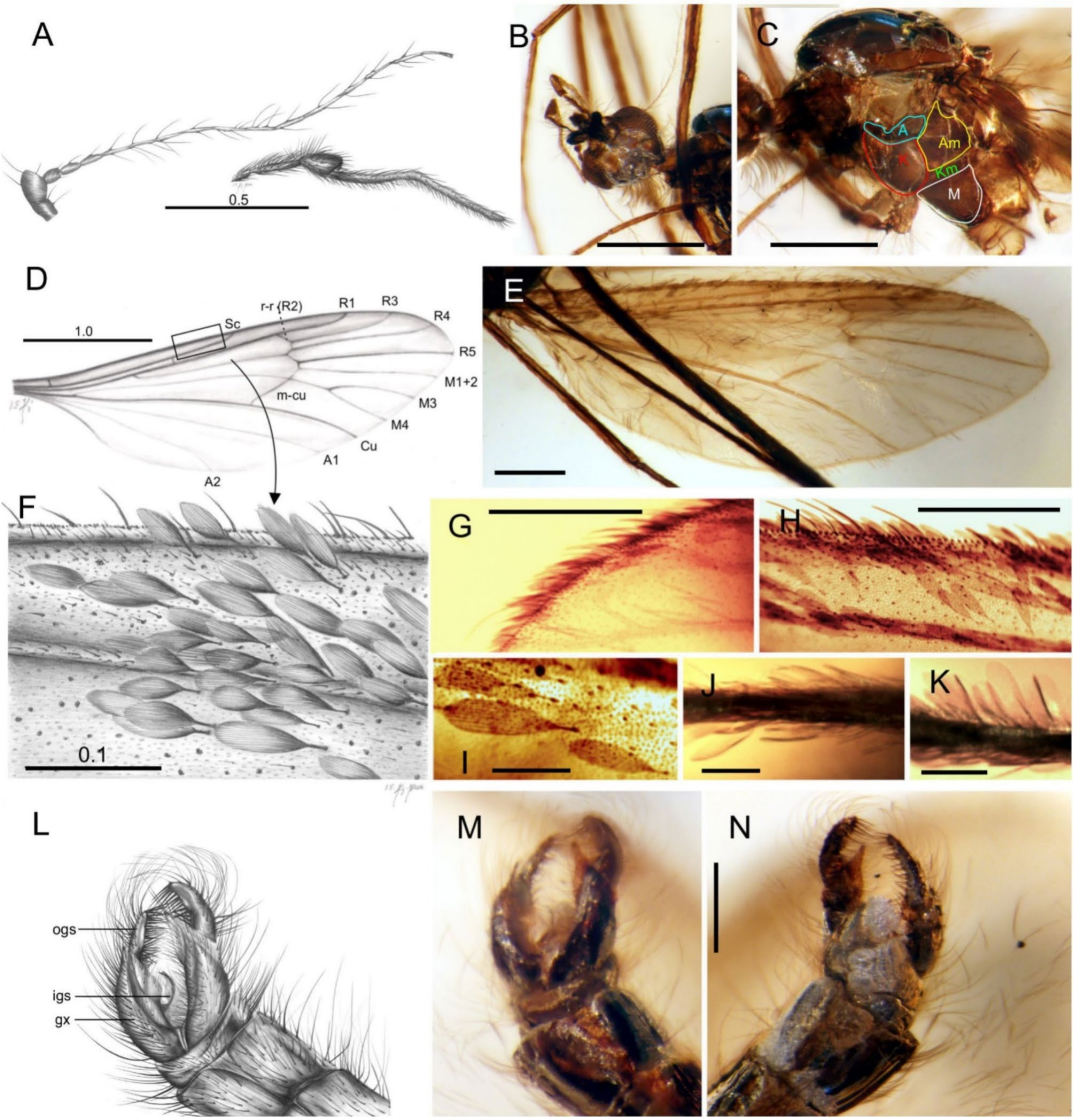
*Maietta hoffeinsetta*, n. sp., holotype No. HT♂, CCHH#1651-2 / SDEI = Dip-00436 - Baltic amber: **A**, reconstruction of antenna and palp; **B**, head, ventral view; **C**, thorax with borders of sclerites highlighted; **D**–**I**, wing: venation (**D**), distribution of scales (**E**), reconstruction of scales (**F**) on fragment of upper costal margin (marked in D), **G**, scales on distal margin, **H**, on costal margin, **I**, scales magnified showing longitudinal ribs; **J-K**, scales on mid an fore femur, respectively; **L-N**, male genitalia: reconstruction and photo of ventral side (**L, M**, respectively), photo of dorsal side (**N**). (A, anepisternum; Am, anepimeron; gx, gonocoxite; igs, inner gonostyle; K, katepisternum; Km, katepimeron; M, meron; ogs, outer gonostyle). Scale bars [mm]: A–C, E: 0.5; D: 1.0; F, I–K: 0.1; G–H, M,N: 0.2

The specimen is entirely preserved, except for the distal part of one fore leg (for habitus see Supplementary Figure S1). Head: ocelli absent; eyes large, oval, bare (no bristles between ommatidia); antennae (Fig. 1A) very thin and 2× longer than palpi, probably 16-segmented, with large pedicel, first flagellomere short and thick, with subsequent flagellomeres longer and thinner toward the end; borders between flagellomeres are poorly recognizable beside first three segments. Palp (Fig. 1A, B): third segment is greatly enlarged and the fourth is extremely long and thin, twice exceeding combined length of preceding palpomeres. Wing oval, 3.7 mm long, widest in mid portion (1.2 mm), anal angle well developed. Venation (Fig. 1D): Sc short, ending just before mid wing; cross vein sc-r in proximal third of wing; Rs somewhat longer than R4; R1 short, terminating in costal wing margin and opposite 2/3 of R3; cross vein r-r (R2) slightly curved, in oblique position, far before end of R1, at level of cross vein r-m and just behind fork of R3 + 4; only three long medial veins present: M1 + 2, M3 and M4; d cell open by atrophy of cross vein m-m; cross vein m-cu very long and strong, positioned in fork of Mb into M1 + 2 and M3 + 4; A1 very delicate and poorly discernible; A2 conspicuous, slightly wavy. Upper sections of the costa, subcosta, and membrane in subcostal sector are covered with microtrichia, macrotrichia and scales. Scales on costal fringe are lanceolate, in two sizes (Fig. 1F-G), those in radial field are oval, with longitudinal ribs, attached to veins and the membrane by long, thin petioles (Fig. 1F, H); some scales became detached from wing and are preserved around it. Thorax: large katepisternum, anepimeron, and meron, all three of comparable size (Fig. 1C).

#### Legs

distal segments are covered over entire length with scales similar to those on wings, more or less adpressed to leg surface; largest and most rounded are present on femora (Fig. 1 J-K); tarsi bear more slender scales, adpressed to surface. Tibial spurs absent. Male: hypopygium (Fig. 1L–N) covered with long bristles and setae; sternite IX narrow; gonocoxite massive and long, at ventral side extended into a sharp, triangular process; gonostyle two-branched, outer branch fleshy, little shorter than gonocoxite, curved inwardly, mesal face with a comb of very strong bristles; inner branch in shape of a narrow sickle. Female unknown.

### Comparison of fossil and recent *Maietta*

**Fig. 2:**
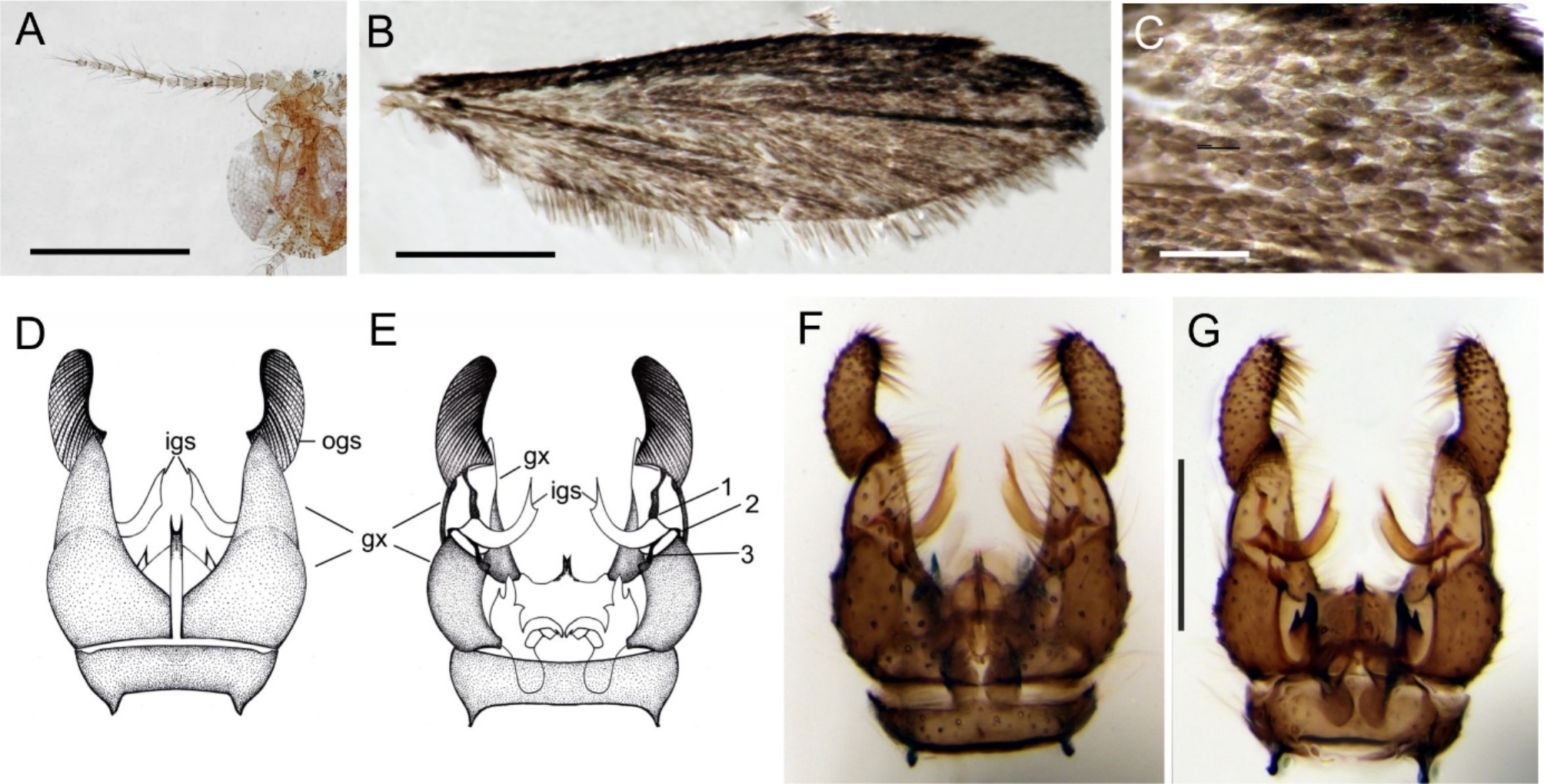
*Maietta trimedia* Alexander, 1967, recent species: **A**, head and antenna; **B**, wing; **C**, density of scales on wing; **D-G**, male genitalia: explanatory drawings of ventral and dorsal view (**D-E**, respectively), photos of ventral (**F**) and dorsal views (**G**). Abbreviations: 1, 2, 3, three tendon-like connections: 1, between inner and outer gonostyle; 2, 3, between inner gonostyle and gonocoxite; other abbreviations as in Fig. 1. Scale bars [mm]: A: 0.5, B: 1.0; C, F, G: 0.2

The differences between the Eocene species *M*. *hoffeinsetta*, n. sp., and recent congeners listed in the diagnosis show that, apart from the wing scale cover, they concern antennae and palpi (detailed morphology of six recent species in [34]). In the Eocene species the antennae were long and filiform, in recent species their length is much shorter (Fig. 2A), although the number of flagellomeres is the same. Palpi have undergone transformation toward further elongation of the last palpomere.

Although the fossil and recent species are separated by 40 Mya of evolution, male genitalia in the entire genus are nearly identical in their external anatomy (compare Fig. 1 L–N for *M*. *hoffeinsetta* and Fig. 2 D–G for recent *Maietta*). Moreover, this morphology is unique among the Limoniidae in details of connection between the gonocoxite and gonostyle. A thin and sickle-shaped inner branch of gonostylus is distinctly shifted from the outer branch and connected with it only by a long, tendon-like stripe (marked 1 in Fig. 2 E), while two other similar stripes (marked 2 and 3 in same figure) connect the inner branch to the gonocoxite. In the fossil species the genitalia are visible only in ventral view, as the large gas bubble obscures partially the dorsal view (Fig. 1 N), but the position of a sickle-shaped process is exactly as in the recent species, protruding halfway from behind the gonocoxite (Fig. 1 L, M). The spatial separation of both branches of gonostyles is exceptional between limoniids, and probably expresses the functional fit to the female genitalia which are also of unusual shape in all recent species: ovipositors’s cerci are small and circular [34]. The female of *M*. *hoffeinsetta*, n. sp. remains unknown, but the similarity of male genitalia in fossil and recent congeners allows to expect finding the female with transformed cerci.

The wing venation in the genus is uniform and characteristic by the vein R2, which is curved, positioned deeply in the wing, and not in the usual vertical position of a cross-vein r-r as in most Limoniidae, including the genus *Amphineurus* Skuse whose venation is otherwise most similar to *Maietta* [38]. In recent species of *Maietta* the vein R2 is longer than in its fossil predecessor, *M*. *hoffeinsetta*, n. sp. The six recent species differ by the degree of this elongation [34]. The medial field is characteristic by its reductions: open discal cell and veins M1 + 2 fused over entire length into M1 + 2; cross-vein m-cu is exceptionally long.

**Fig. 3:**
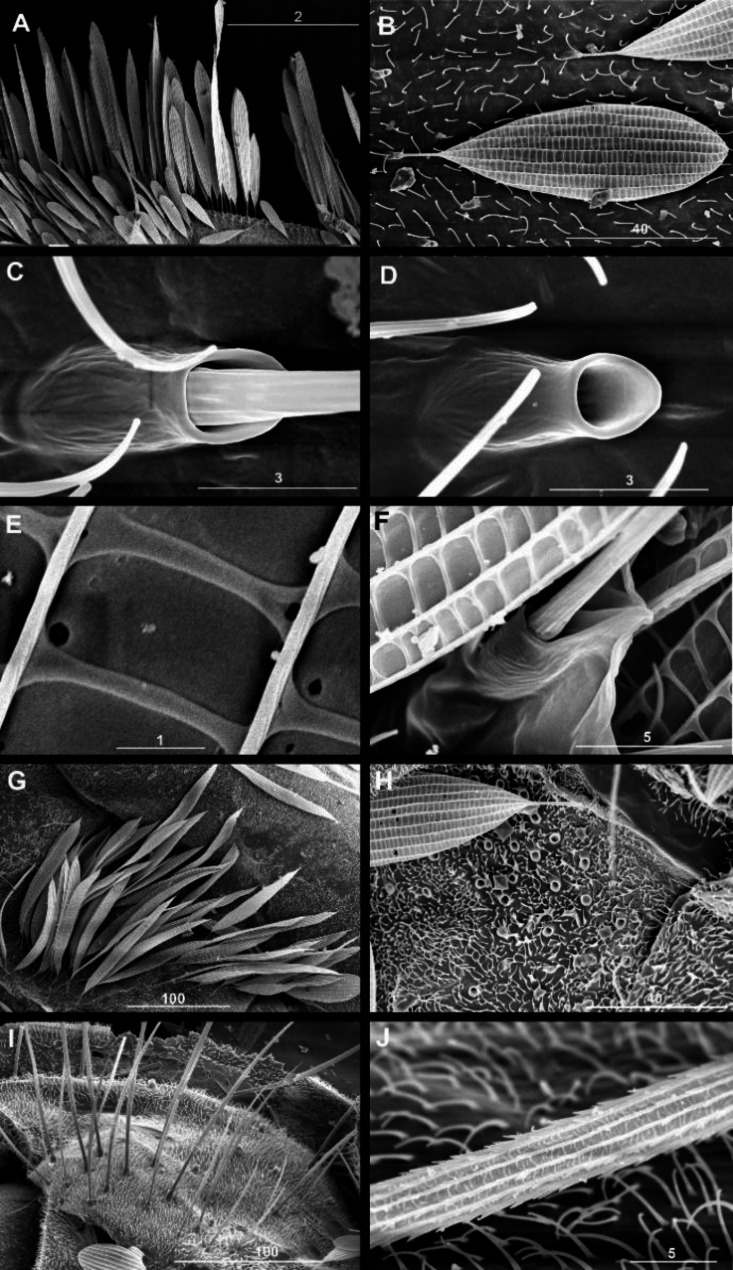
*Maietta trimedia* Alexander, 1967, recent species, scales, macro-and microtrichia on wing (A–E) and thorax (F–J): **A**, wing margin; **B**, single scale anchored in wing membrane set with microtrichia; **C**, pocket of scale; **D**, empty pocket after the scale had been lost; **E**, detail of transverse and longitudinal ribs of scale; **F**, pocket of scale on thorax; **G**, brush of scales on katepisternum; **H**, loose scale and several empty pockets among microtrichia; **I**, brush of macrotrichia on metanepisternum; **J**, single macrotrichium with longitudinal and cross ribbing. Scale bars in µm: A, G, I: 100; B, H: 40; C, D: 3; E: 1; F, J: 5

The most spectacular difference between the Eocene and recent time is the development of scale cover on the wings. While in *M*. *hoffeinsetta* the scales are restricted to the costal and partially radial field (Fig. 1 E), in recent species the scales densely cover entire wing in overlapping layers, thus superficially reminding wings of a moth (Fig. 2 B, C; also [34]). The scales are oval, approx. 0.15–0.2 mm long (Fig. 1 I for fossil and Fig. 3B for recent), and bear longitudinal ribs; in recent species these ribs are interconnected by crossribs (Fig. 3E, F); in fossil species the presence of crossribs cannot be confirmed because of optical distortion caused by a layer of amber. The crossribs are present also in macrotrichia (Fig. 3J). The scales which form a fringe are more narrow, longer and lanceolate (Fig. 1 G, H, and 3 A, respectively), although in the fossil species the fringe is restricted to the upper costal to radial margin, while in recent species the fringe goes aroung entire wing. The scales are present on legs, and in recent species also on the thorax; especially on the anepisternum, the scales are very long, lanceolate; their crossribs are still well-recognizable (compare Fig. 3 F). The scales are anchored in elevated narrow sockets (Fig. 3 C, D); empty sockets are numerous on the wing and thorax (Fig. 3 H) thus documenting earlier loss of scales. Generally, the scales in *Maietta* do not differ from those observed in other Diptera, and from the simplest scales in the Lepidoptera, as those in the Micropterigidae [1–3]. The elevated sockets in which the scales are anchored (Fig. 3 C, D, F) also belong to the groundplan in the Diptera [18] and Lepidoptera [2].

The macrotrichia reside in sockets on wing veins and membrane, and are present in groups on thorax and other body parts (Fig. 3 I). Microtrichia are very small, more or less curved, and distributed over the wing membrane and all body parts (Fig. 3B, J).

## Discussion

### Evolution of scales on wings in the Diptera

#### Scales appeared first on legs, then on wings

**Fig. 4:**
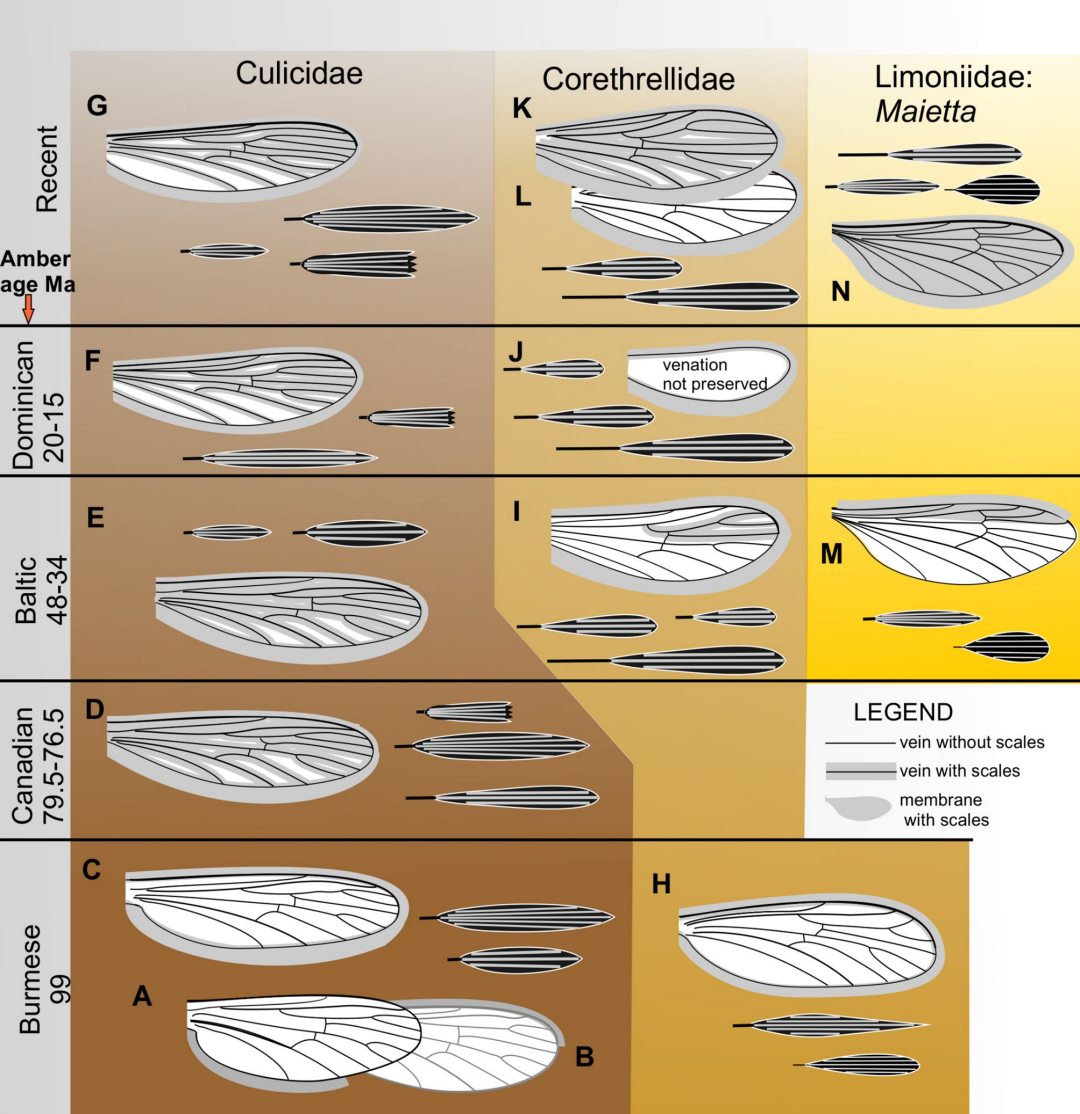
Development of scale cover in wings of fossil Diptera. Dipteran families with scaled wings through epochs; representative species preserved in fossil resins. Basic shapes accompany relevant wings; width of lower costal fringe is proportional to wing size. **A, B,** *Burmaculex antiquus*, costal fringe preserved fragmentarily in two females (Burmaculicinae); **C**, *Burmaculex burmanicus*; **D**, *Paleoculicis minutus*; **E**, *Cocquilletidia* sp.; **F**, *Culex malariager*; **G**, *Culex pipiens*; **H**, *Corethrella andersoni*; **I**, *Corethrella miocaenica*; **J**, *Corethrella prisca*; **K**, *Corethrella kipferi*; **L**, *Corethrella oppositophila* (**K**, **L**, recent representatives of Corethrellidae with costal fringe only and with veins covered with scales, respectively); **M**, *Maietta hoffeinsetta*, sp. nov.; **N**, *Maietta trimedia*. Chronology axis is not to geological scale

The oldest dipterans with scales are preserved in Cretaceous Burmese amber and represent a lineage ancestory to mosquitoes, Burmaculicidae [17, 29]. Subsequent, younger inclusions of mosquitoes allowed to trace the evolution of the scale wing cover in these dipterans. Borkent and Grimaldi [17, 29] postulate that about the Lower Cretaceous the scales began to differentiate from the macrotrichia, and first appeared on the legs, then on the wing margin, and later still, during the upper Cretaceous, on the wing veins. Scale shape evolved from narrow and lanceolate to the various shapes now present even on same wing, as also occurs in recent mosquitoes. Similar evolutionary sequence is indicated by fossils of another culicomorph group, the Corethrellidae [39–41]. Supporting data on dipteran fossils are summarized in Table 1 and presented in Fig. 4.

The same developmental track is observed on comparing the Eocene and recent species of the limoniid genus *Maietta*: the Eocene species has dense scales on legs, but in wing they are scarce and restricted to the subcostal field and wing margin, while recent species of *Maietta* have wings, legs and thorax densely covered with variegated scales (Fig. 3; also [34]).

**Table 1:** Records on scale cover in wings and legs of fossil Diptera of the Corethrellidae, Culicidae, Ceratopogonidae and Limoniidae. +/-, presence/absence of scales. References to age of fossil resins: Lebanese amber [42]; Burmese amber [21]; Canadian amber [43], Baltic amber [35]; Dominican amber [44]

Taxa (Fig. in this paper)	Sex	Age of amber	Scales			Reference
			on legs	lanceolate around margin	on veins	
**Corethrellidae**						
*Corethrella (Fossicorethrella) cretacea*	1 ♂	Lebanese (135–125 Ma)	-	-	-	[40]
*Corethrella andersoni* (Fig. 4H)	1 ♀	Burmese (98.79 Ma)	+	+	-	[41]
*Corethrella patula*	1 ♂	Burmese (98.79 Ma)	+	-	-	[45]
*Corethrella (Nothocorethrella) miocaenica* (Fig. 4I)	1 ♂	Baltic Bitterfeld (48–34 Ma)	-	+	-	[46]
*Corethrella prisca* (Fig. 4J)	1 ♂	Baltic Bitterfeld (48–34 Ma)	-	+	+ (radial veins)	[39]
*Corethrella nudistyla*	1 ♂	Dominican (20–15 Ma)	-	+	-	[39]
*Corethrella*, e.g.:		recent	-/+	-/+	-/+	[47]
*C. oppositophila* (Fig. 4L),	♂♂, ♀ ♀	recent	-	+	+	[48]
*C. kipferi* (Fig. 4K)	♂♂, ♀ ♀	recent	-	+	-	[49]
**Culicidae**						
*Burmaculex antiquus* (Fig. 4A, B))	2 ♀	Burmese (98.79 Ma)	+	+	-	[17, 29, 50]]
*Burmaculex burmanicus*	1 ♀	Burmese (98.79 Ma)	+	+	- (fusiform setae)	[32, 50]
*Burmaculex edwardsi*	1 ♀	Burmese (98.79 Ma)	+	+	-	[50]
*Burmaculex harbachi*	1 ♀	Burmese (98.79 Ma)	+	+	-	[50]
*Burmaculex porczynskii*	1 ♂, 1 ♀	Burmese (98.79 Ma)	+	+	-	[50]
*Paleoculicis minutus* (Fig. 4D)	1 ♂	Canadian (79.5–76.5 Ma)	+	+	+	[31]
*Culiseta gedanica*	1 ♂	Baltic (48–34 Ma)	+	+	+	[33]
*Ochlerotatus serafini*	1 ♂	Baltic (48–34 Ma)	+	+	+	[33]
*Coquillettidia sp.* (Fig. 4E)	1 ♂	Baltic (48–34 Ma)	+	+	+	[33]
*Culex erikae*	1 ♂	Baltic (48–34 Ma)	+	+	+	[51]
*Culex malariager* (Fig. 4F)	1 ♀	Dominican (20–15 Ma)	+	+	+	[30]
Several species e.g. *Ochlerotatus* sp. (Figs. 4G and 5)	♂♂, ♀ ♀	recent	+	+	+	this paper
**Ceratopogonidae**						
Several species	♂♂, ♀ ♀	Cretaceous ambers (145–66 Ma): San Just, El Soplao, Taimyr, Burmese, Canadian	-	-	-	[52]
Several species	♂♂ ♀ ♀	Indian Eocene (54.5 Ma)	-	-	-	[53]
Forcipomyia (Lepidohelea) domibicolor*	9 ♂, 11 ♀	Dominican (20–15 Ma)	-	+ (membrane of radial cells)	-	[54]
32 species	239♂ 442♀	Dominican (20-15 Ma)	-	-	-	[54]
*Forcipomyia*	♂♂, ♀ ♀	recent	-/+	-/+ (on wing membrane)	-/+	[55]
**Limoniidae**						
*Maietta hoffeinsetta n. sp.* (Fig. 1)	1 ♂	Baltic (48-34 Ma)	+	+	+ (on radial sector)	this paper
6 species of *Maietta* e.g. *M. trimedia* (Figs. 2B-C and 3)	♂♂, ♀ ♀	recent	+	+	+	[34], this paper

#### Scales appeared late in evolution of the Diptera

The oldest evidences of butterfly scales are known from the beginning of the Triassic (Hettangian [22]). It is postulated that this adornment appeared in, or even before the earliest stages of butterflies’ evolution in the beginning of the Triassic [19].

Although the oldest Diptera are also documented from the early Triassic [28, 56, 57], the oldest specimens (mosquitoes) with scales are not known before the Cretaceous. Quite contrarily, the macrotrichia which are homologous to the scales, are ubiquitous in wing imprints beginning from the Jurassic (e.g., Trichoceridae [58]; Tanyderidae [59], and on nearly all wings of Diptera embedded in fossil resins beginning from the early Cretaceous, e.g.: Psychodidae in Baltic, Lebanese, France and Taimyr amber [60–63], Limoniidae in Burmese amber [64], Psychodidae in same resin [65], in Fushun Eocene amber [66], and in Baltic amber [67]). Summarizing, the present data available indicate an early evolution of scales in the Amphiesmenoptera (Triassic), while in the Antliophora—and only the Diptera —the scales appeared in the Cretaceous, although their precedessors, the macrotrichia, have occurred on wings since the early Jurassic at least.

#### Cross ribs occur both in the macrotrichia and in the scales in the Diptera

**Fig. 5:**
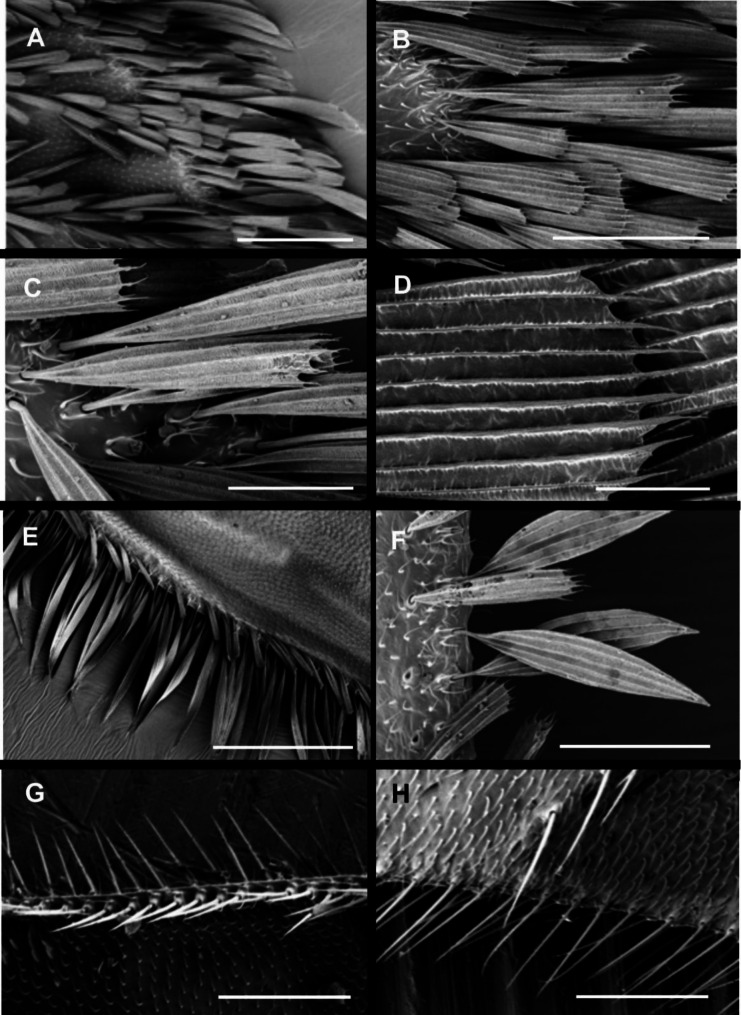
Scales, macro- and microtrichia on wing in recent mosquito *Ochlerotatus* sp.: **A-D**, broad, spade-shaped scales on veins, subsequent magnifications; **E-F**, lanceolate scales on wing margin; **G**, macrotrichia on wing margin; **H**, macro-and microtrichia. Scale bars in µm: A, D: 10; B: 50; C: 20; E: 200; F: 40; G, H: 100

Macrotrichia (bristles), microtrichia (setae, hairs) and scales are homologous structures. It has been documented that the scales evolved from the macrotrichia, and the ontogenetic development of a scale shows its transition from rounded to a flat shape [68]. Herein we found that in *Maietta* not only the scales, but also the macrotrichia bear both the longitudinal and cross ribs (compare Fig. 3J). The longitudinal ribs in macrotrichia were previously documented in various Diptera [18] but the magnification applied was not sufficient to document the cross ribs. The cross ribs found in mosquitoes’ macrotrichia appear very delicate and dense, of the herring bone type (Fig. 5C, D, this paper; [68]).

A similar cross ribbing in macrotrichia is visible also in *Psychoda* (Psychodidae [69]). Therefore it seems plausible that in the Diptera both kinds of ribs are ubiquitous, in both the macrotrichia and the scales, thus morphologically corroborating their common genetic background. This finding is important in view of the currently proposed concept of scales evolution in the Amphiesmenoptera [19]. Based on the scales in the Tarachoptera (an extinct order, and a sister group to the Trichoptera + Lepidoptera), and those in the family Lepidochlamidae (basal to the Trichoptera), the absence of cross ribbing in the scales was defined a plesiomorphic state, present only in these two extinct stem groups of the Amphiesmenoptera [19]. This plesiomorphic state has not previously been reported in Diptera.

### Role of scales in *Maietta*: thermoinsulation?

*Maietta hoffeinsetta*, n. sp., lived in northern hemisphere on the territory of recent Europe, and inhabited subtropical forests soaked with water, an environment where some still unidentified gymnosperm trees [70] exuded abundant resin to be later fossilized into Baltic amber. The distribution of *Maietta* in the Eocene is unknown, but it had to be much wider than Europe, regarding present localities of this genus in the antipodes of South America. In Eocene the climate in Europe and South America was warm and similar, according to climate maps of Eocene [71]; see Supplementary file S2. Since that time, the genus became extinct in wide regions, with the only known extant locality in Chile, and it lives now in moderate to cool climate. Its habitats are coastal temperate rainforests characterized by a combination of altitude variations, oceanic influence, and mainly high precipitation, which form a characteristic mosaic sufficientto support diversity in this forest type [72]. The species of *Maietta* may appear at altitude c. 1000 m where the winters are frequently snowy, and some localities (e.g., Aysen) have mean temperatures comparable to those in Central Scandinavia (for more information see Supplementary file S2).

Thus it is possible that the post-Eocene necessity of adaptation to a cooling climate was a trigger of the evolution of rich scale cover on both wings and body. Such a scenario is suggested by the thick cover of wing scales that evolved after the warm Eocene. The scales are known to make an excellent insulator [8, 9] and could enable the descendants of Eocene species of *Maietta* to survive in cooler climate. It is easily realizable that, in cold air, the haemolymph circulating in the wing veins may significantly lower the body temperature, and the extra insulation provided by scales may slow or prevent this process, thus enabling or facilitating flight. A further indication of adaptation to cool climate are the short antennae composed of thick and short („plump”) antennal flagellomeres in recent species of *Maietta* (Fig. 2A; also [34]. A similar phenomenon of shorter antennae in cold climate, observed in several species, has been described in winter flies (Trichoceridae) [58].

### Climatic preferences of recent congeners: are they reliable indicators of paleoclimate?

The disjunct distribution between Eocene and recent times which is characteristic for *Maietta* is known since long in other insects, e.g.: micropterigid butterfly *Baltimartyria*, with Baltic amber being the only known locality in northern hemisphere [72]; termites Kalotermitidae (recently Mediterranean, south Africa, south Australia, New Zealand, Chile [73]; Diptera, Ceratopogonidae: genus *Macrurohelea* (Chile and southeast Australia [74]. Among the beetles, the Staphylinini related to those in Baltic amber are now outside the Palaearctic, and encompass both thermophilic and moderate taxa [75, 76].

The paleoclimate of Baltic amber forest poses challenges to reconstructions based on climatic preferences of recent congeners of insects in Baltic amber. The data show that amber forest ecosystem consisted both of thermophilic and temperate organisms. This discrepancy was a matter of debate since more than 100 years (e.g [77]. , , under the term of the „Wheeler’s dilemma” following the early observations of Wheeler [78] on ants in Baltic amber. The possible three explanations were discussed [75, 76, 79], according to which this composition of fauna may reflect:

1. changes of climate in Eocene during long durance of amber producing forests,.
2. spatial divergence in various parts of forests wide-spread across warmer lowlands and cooler mountains,.
3. equable temperature seasonality, with temperate summers and mild winters which allowed to survive thermophilic organisms.

The latter explanation seems most probable [76], and the argument may be added here that the seasonal equability in Baltic amber forest is expected in Eocene Europa composed of islands, that is, under immediate moderating influence of the ocean.

If our hypothesis on post-Eocene adaptation to cooler climate in *Maietta* is correct, then changes in climatic demands in a particular genus may occur more often and the reconstruction of paleoclimate of Baltic amber forest based on recent descendants’ preferences may not always be justified. On the other hand, the climatic tolerance in insect species, and the more so, in genera, seems to be much underestimated. For instance, some species of winter gnats (Trichoceridae) show amazing spectrum of adaptational possibilities [80]: e.g., *Trichocera saltator* Harris, *T. regelationis* L., and *T. hiemalis* DeGeer occur from arctic latitudes far beyond the Polar Circle (for Taimyr Peninsula, c. 75^o^N [81]) to north Africa (c. 30^o^N; [82]), and are actively flying in temperatures from 0^o^C to over 20^o^C.

## Conclusions

We have documented the first finding of fossil Diptera with scales on wings in a taxon other than Culicidae. This specimen, embedded in Baltic amber, belongs to a recent, relict genus *Maietta* in the Limoniidae, now extant only in Chile. The distribution of scales, dense on legs and scarce on wing, indicates parallel evolution of scale cover in two remote families: Culicidae and Limoniidae. The main stages of this evolution included, in order, the appearance of scales on legs, around the wing margin, and encompassing entire wing.

The dense cover of scales on wing and body, and shortened, thickened antennae are the main differences between recent males of the genus and those from Baltic amber, and may indicate adaptation in this genus to cooling down the climate after the Eocene, and far outside Europe, in the antipodes of South America.

## Electronic supplementary material

Below is the link to the electronic supplementary material.

Supplementary material 1

## Data Availability

Data supporting the findings of this study are available within the article and its supplementary material.
