# Supplementary material for: Evolution of wing scales in Diptera documented by fossils
Source: Zoological Lett. 2024 Dec 30;10:22. doi: 10.1186/s40851-024-00244-x (PMC11687164; doi:10.1186/s40851-024-00244-x)
Supplement: Supplementary file 1 — Supplementary material 1 [file 40851_2024_244_MOESM1_ESM.docx]

**Supplementary file to**:

Evolution of wing scales in Diptera documented by fossils

Ewa Krzemińska^1^*, Wiesław Krzemiński^1^, Iwona Kania-Kłosok^2^, Jadwiga Stanek-Tarkowska^4^, Kornelia Skibińska^1^, and Daubian Santos^5^

*^1^ Institute of Systematics and Evolution of Animals, Polish Academy of Sciences, Sławkowska 17, 31-016 Kraków, Poland; e-mail: ekrzeminska9@gmail.com*

*^2^* *Department of Biology, University of Rzeszów, Zelwerowicza 4, 35–601 Rzeszów, Poland; e–mail: ikania@univ.rzeszow.pl*

*^4^Institute of Agricultural Sciences, Land Management and Environmental Protection, University of Rzeszów, Zelwerowicza 8B, 35-601 Rzeszów, Poland; e–mail: dyrektor.ir.cn@ur.edu.pl*

^5^ *Universidade Federal do ABC, Centro de Ciências Naturais e Humanas, Av. dos Estados, 5001. Bairro Bangu, 09210-580, Santo André, SP, Brazil*


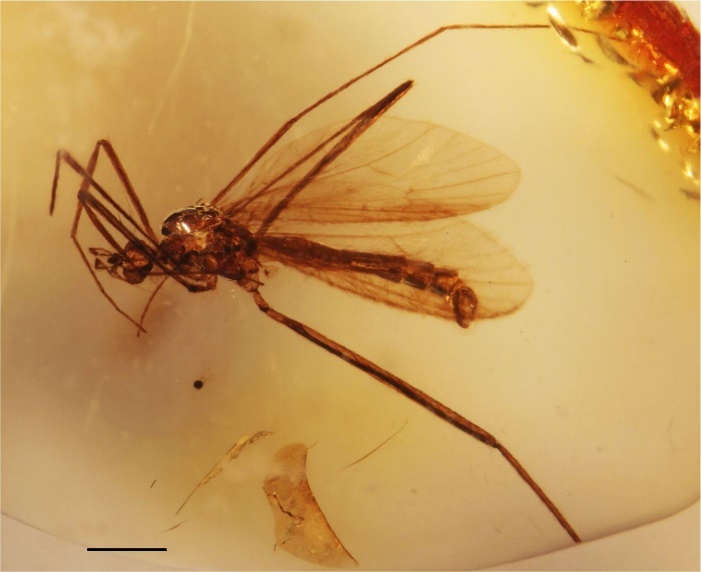


Figure S1. Habitus of *Maietta* *hoffeinsetta*, n. sp., holotype. Scale bar 1 mm.

S2. Climate in past and recent localities of *Maietta*


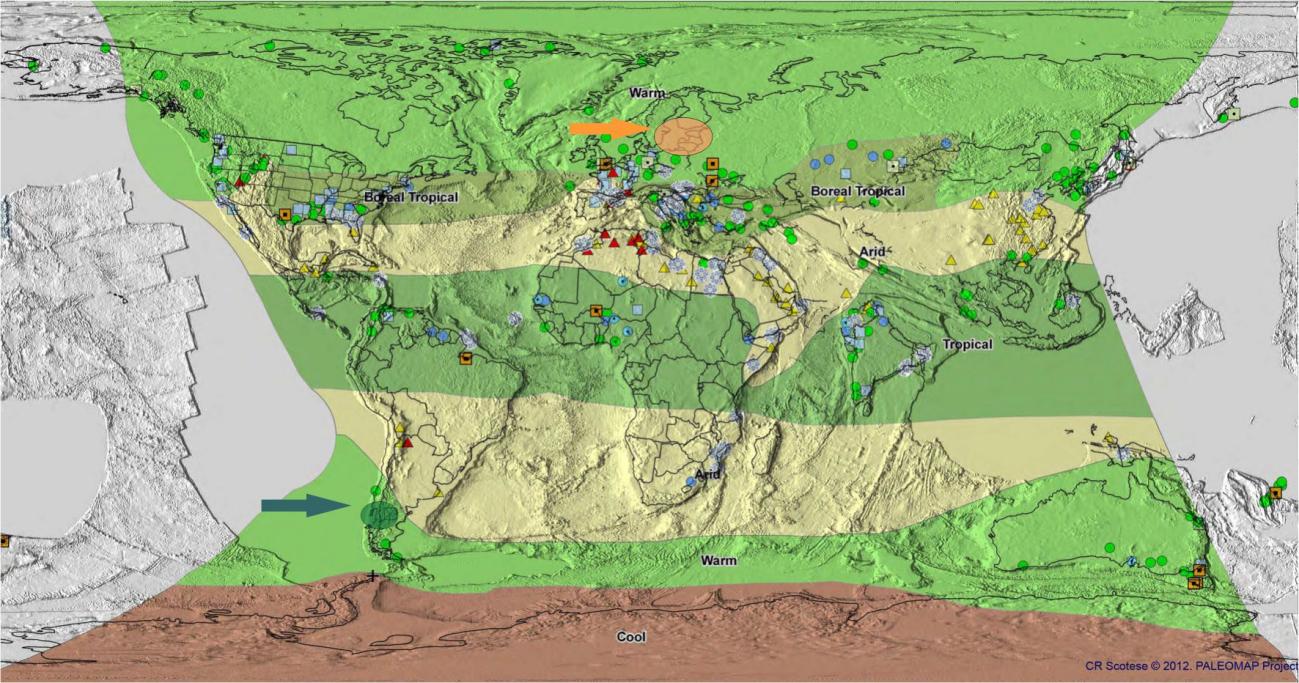


Figure S2A. Climate in Eocene in the region of Baltic amber formation orange arrow) and in southernmost region of South America (green arrow) was similar, and has been described as warm [71]. Orange arrow: Baltic amber presumed locality of *Maietta* (*Hoffeinsoneura*) *christelae*, n. sp.; green arrow: recent locality of extant species of *Maietta*.


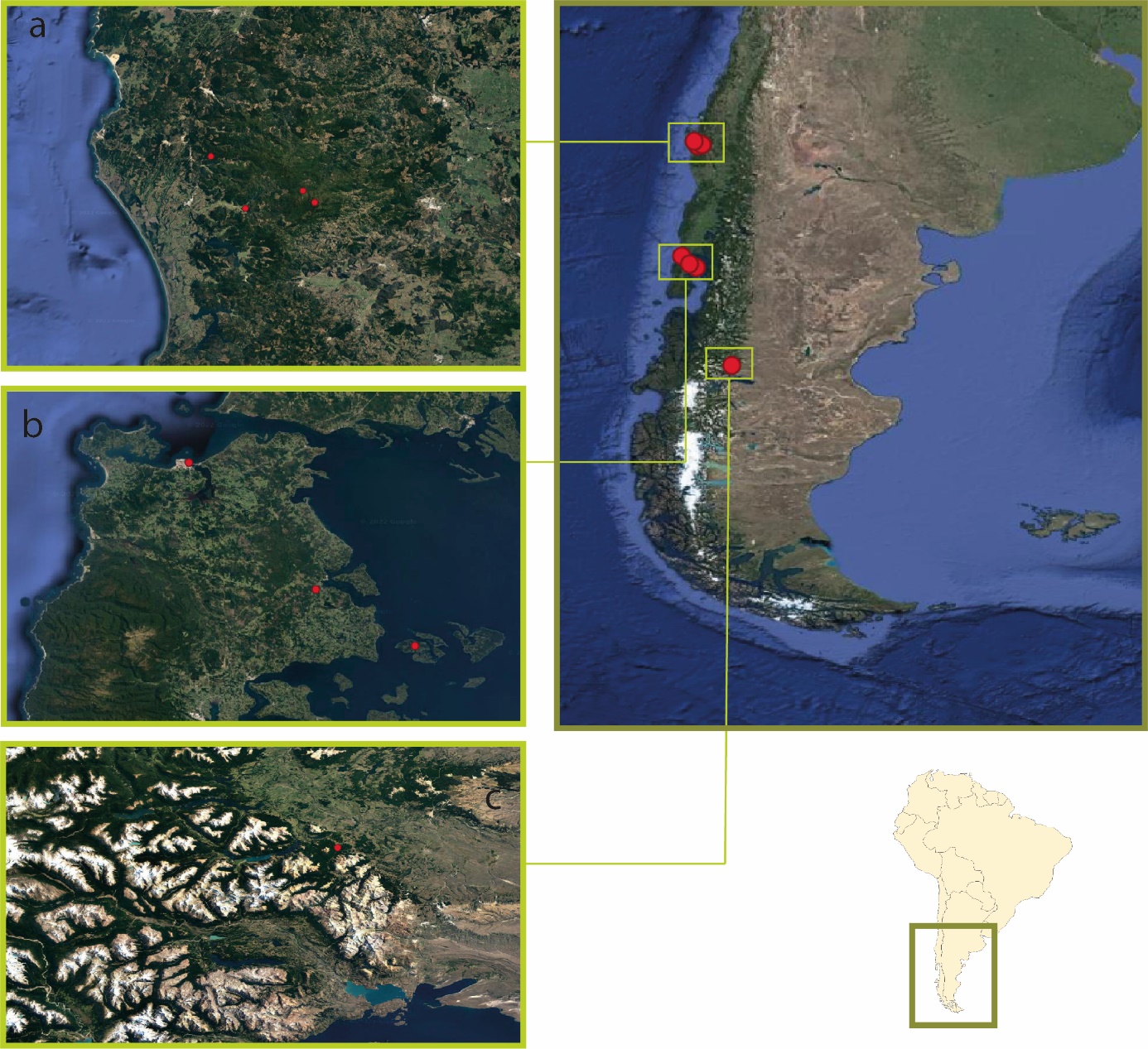


Figure S2B. Geographical distribution of *Maietta* in South America: a) Nahuelbuta forest; b) Chiloé evergreen forest; c) Aysén.

Nahuelbuta: Specimens are collected near water bodies or islands, some of them at significant altitudes (over 1km), where winters are frequently snowy. The Nahuelbuta forest: Meteorological data are lacking for higher elevations, but precipitation is probably greater than that recorded at sea level, with frequent winter snowfall above 1000 m. The minimum mensal average temperature recorded in Concepcion was 8.7ºC (July 1998 and 1999), and the maximum mensal average temperature was 16.6ºC [83, 84].

Isla Chiloe evergreen rain forest. The Chilean coastal range: a vanishing center of biodiversity and endemism in South American temperate rainforests. Snow does not commonly occur in these locales. Chiloe evergreen forests: an annual precipitation between 5000 and 6000 mm [84]. Mean monthly temperatures at sea level vary between 6ºC in July and a maximum of 16ºC in January.

Aysen, the southernmost and coldest locality: average temperature for coldest month, July, from –3 to +3^o^C; for warmest, January, 7–17^o^C (source: https://en.climate-data.org/south-america/chile/region-aysen-del-general-carlos-ibanez-del-campo/valle-simpson-876092/#temperature-graph). This climate seems similar to southern Scandinavia; for example, Karlskrona, southern Sweden: Jan. –1.5 to +3 ^o^C, July 15–17 ^o^C (source: https://en.climate-data.org/europe/sweden/blekinge-laen/karlskrona-6274/#temperature-graph).
